# Modular iodinated carboxybetaine copolymers as charge-sensitive contrast agents for the detection of cartilage degradation

**DOI:** 10.1016/j.mtbio.2024.101302

**Published:** 2024-10-26

**Authors:** Patrick Weber, Annalena Maier, David Fercher, Maryam Asadikorayem, Marcy Zenobi-Wong

**Affiliations:** Tissue Engineering + Biofabrication Laboratory, Department of Health Sciences and Technology, ETH Zürich, Otto-Stern-Weg 7, 8093, Zürich, Switzerland

**Keywords:** Zwitterionic polymers, Contrast-enhanced computed tomography, Cartilage, Osteoarthritis

## Abstract

Accurately assessing cartilage tissue degradation is a big challenge in osteoarthritis (OA) research, as histology only provides information about a 2D tissue section, and currently available contrast agents for tomographic evaluation suffer from low specificity. In this study, we present a modular platform based on zwitterionic carboxybetaine (CBAA) to create multivalent polymeric contrast agents for x-ray computed tomography (CT) with high specificity towards the anionic glycosaminoglycans in the cartilage tissue. By copolymerizing CBAA with different ratios of anionic and cationic iodinated comonomers, we created a library of polymers with net charges ranging from strongly anionic to strongly cationic. The polymers were applied onto osteochondral plugs with different degradation states and the resulting CT images compared to histological stainings. In healthy tissues, the bulk contrast enhancement was strongly correlated with polymer charge, with cationic polymers reaching a 2-fold stronger contrast compared to established small molecule contrast agents. While a further increase in cationic charge slowed the penetration, it increased the polymer's specificity, thereby enabling the most cationic polymer C40 (40 mol% cationic iodinated comonomer) to discriminate accurately between tissues treated with IL-1β for 0, 1, 2 and 3 weeks. Moreover, this polymer also showed a strong local specificity, visualizing local differences in GAG distribution with significantly increased accuracy compared to the controls. Our polymer contrast agents show the importance of multivalency and charge control for the accurate, volumetric detection of GAGs in the cartilage tissue and paves the way towards new contrast agents in- and outside of the clinic.

## Graphical abstract

**Figure:**
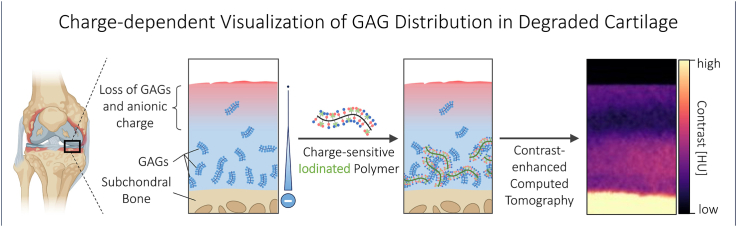

## Abbreviations

ANIC: Anionic iodinated comonomer
ATIIPA: 3-Amino-2,4,6-triiodoisophthtalic acid
CATIC: Cationic iodinated comonomer
CBAA: Carboxybetaine acrylamide
CECT: Contrast-enhanced computed tomography
CT: Computed tomography
DMA: Dimethylacetamide
DMSO: Dimethyl sulfoxide
ECM: Extracellular matrix
GAG: Glycosaminoglycan
IL-1β: Interleukin-1 beta
MMP: Matrix metalloproteinase
OA: Osteoarthritis
TLC: Thin layer chromatorgraphy
THF: Tetrahydrofurane

## 1 Introduction

Osteoarthritis (OA) is a degenerative joint disease that affects more than 500 million individuals worldwide [1]. Despite its significant impact on patients’ quality of life and the high costs for healthcare systems, current treatments primarily focus on pain relief and anti-inflammatory mechanisms, with total joint replacement at the late stage. The lack of effective tools to assess cartilage degeneration presents significant challenges in both research and (pre-)clinical diagnosis.

The extracellular matrix (ECM) of articular cartilage is organized into distinct zones, each with unique structural and biochemical properties; together they give cartilage its excellent mechanical properties [2]. The content of glycosaminoglycans (GAGs) increases progressively from the superficial to the middle and deep zones, creating a gradient of negative charge [3,4] (Fig. 1A). In OA, matrix-degrading enzymes are overexpressed, leading to a loss of ECM starting from the articular surface. The primary enzymes driving this process are aggrecanases and matrix metalloproteinases (MMPs). The former are responsible for degradation of GAGs, whereas MMPs mainly target collagen type II, which increases permeability of the ECM, accelerating GAG loss further through their release. The loss of GAGs leads to an overall change in charge density of the cartilage ECM, making GAG content a promising marker for cartilage degradation [5,6] (Fig. 1A).

**Fig. 1:**
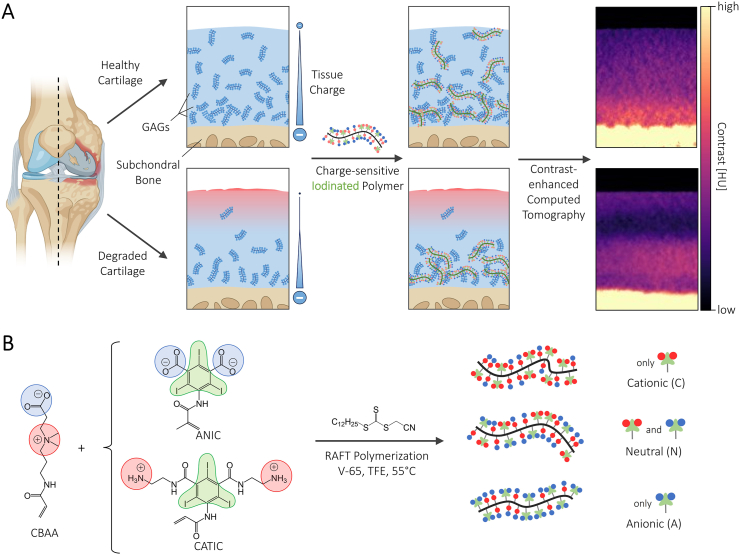
Project overview: A) During cartilage degradation, anionic glycosaminoglycans (GAGs) are lost from the cartilage tissue, leading to changes in zonal charge distribution. By employing iodinated, charge-sensitive zwitterionic polymers that bind to the GAGs, we aim to visualize the local GAG distribution via x-ray micro-computed tomography to assess the degree of degradation within the tissue. B) Polymeric contrast agents were synthesized by RAFT copolymerization between zwitterionic carboxybetaine acrylamide (CBAA) and a combination of anionic (blue) and cationic (red) iodinated comonomers (ANIC/CATIC) to control the net charge of the polymers. Both ANIC and CATIC were synthesized from 3-amino-2,4,6-triiodoisophthtalic acid and contain three iodine atoms (green) per molecule to provide contrast. Figure created with BioRender. (For interpretation of the references to color in this figure legend, the reader is referred to the Web version of this article.)

The most common method to evaluate cartilage degradation is histology. Key staining for cartilage assessment includes Safranin O/Fast Green: this cationic dye binds to anionic GAGs, resulting in a staining intensity that linearly correlates with the GAG content of the sample [7]. Although histology provides detailed information about the local cartilage structure and composition, it fails to capture the whole tissue volume [8]. Histology is furthermore a rather low-throughput analysis technique, as decalcification of the subchondral bone takes several days. Moreover, histology is restricted to *ex vivo* applications, with either biopsies or post-mortem analyses being required. Three-dimensional tomographic techniques, such as magnetic resonance imaging and computed tomography (CT) circumvent these limitations [9]. The use of contrast agents (contrast-enhanced computed tomography, CECT) overcomes the limitations of low attenuation of soft tissues. In addition to merely visualizing the cartilage tissue, the use of charged contrast agents also provides information on the degradation levels [10]. Ioxaglate, a small molecule anionic contrast agent, is repelled by high GAG content but penetrates more when cartilage is degraded and GAGs are depleted [11,12]. Ioxaglate is widely used in clinics, but its inverse charge-targeting requires very high concentrations up to 300 mg iodine/mL [13], increasing the risk for side effects and toxicity. Bajpayee and co-workers were able to improve ioxaglate penetration with the use of the cationic carrier protein Avidin. They found that a weak-reversible binding of the molecules is needed for full-thickness penetration. By comparing the mean contrast in healthy and degraded cartilage, they found that the contrast is significantly reduced in the degraded condition [14,15]. There have been other cationic contrast agents published, including tantalum oxide nanoparticles [16], but the most promising candidate is CA4+ [17]. It is an iodinated, small molecule with four positive charges. Nelson et al. studied CA4+ specificity in an equine defect and regeneration model. They induced chondral defects which they collected either with or without healing time. In both models, they could show that the contrast of the small cationic molecule correlated with the concentration of GAGs present in healthy, degraded and repaired regions of cartilage with a high specificity. However, they used relatively high concentrations of 8 and 24 mg iodine/mL, respectively [18,19].

In our work, we developed a contrast agent based on iodinated, charge-sensitive zwitterionic polymers. Due to the multivalency and increased specificity of these polymers, lower iodine concentrations can be used to achieve even better contrast compared to literature. The modular platform enables the synthesis of polymers with varying charges and affords opportunity to extend their application from purely diagnostic purposes to therapeutic use by conjugation to drugs. The side chains of zwitterionic polymers carry positive and negative charges at equal amounts, which enables high levels of hydration, excellent non-fouling properties and *in vivo* biocompatibility [[20], [21], [22]]. Our polymers are mainly composed of carboxybetaine acrylamide, which has been shown to have a residual positive charge at physiological pH, giving them excellent penetration kinetics into cartilage [23]. To control the net charge of the polymers, we designed and synthesized anionic and cationic iodinated co-monomers (Fig. 1B). We were able to (1) synthesize a library of polymers with charges ranging from cationic to anionic that (2) showed a stronger charge-dependent contrast at a concentration of 1 mg iodine/mL than established small molecule contrast agents, due to multivalent binding. Furthermore, (3) our polymers were able to distinguish between different degradation stages of cartilage in an IL-1β OA model, both with regard to the measured bulk contrast of the tissue and to the changes in local GAG distribution. Overall, our findings illustrate the potential of our polymeric contrast agent to be used for the non-destructive, 3D visualization of the GAG distribution, offering an advanced method to complement histological analysis of cartilage *in vitro*.

## 2 Materials and methods

### 2.1 Chemicals

Unless otherwise stated, all chemicals were purchased from Sigma-Aldrich (CH).

### 2.2 CBAA monomer synthesis

CBAA was synthesized as previously reported [24] (Fig. S1A): 14.9 g freshly distilled N-[3-(Dimethylamino)propyl] acrylamide (95 mmol, TCI Chemicals, JPN) was dissolved in 100 mL of dry THF and cooled to −10 °C. 9.6 g of β-propiolactone (8.4 mL, 134 mmol, 1.4 eq., Acros Organics, USA) was diluted in 25 mL of dry THF and added dropwise over 40 min. After 4 h of continuous stirring, the reaction mixture was kept static at −20 °C overnight. The white precipitate was filtered off on a fritted-glass filter (S4 porosity) and washed with three volumes of cold diethylether to yield 13 g of CBAA (57 mmol, 60 % yield). ^1^H NMR (400 MHz, D_2_O): *δ* (ppm) 6.32 (dd, 1H, COC*H*=CH2), 6.2 (dd, 1H, COCH=C*H*H), 5.87 (dd, 1H, COCH=CH*H*), 3.66 (t, 2H, N-C*H*_2_-CH_2_-COO), 3.48 (m, 4H, NH-C*H*_2_-CH_2_-C*H*_2_), 3.17 (s, 6H, N-(C*H*_3_)_2_), 2.75 (t, 2H, C*H*_2_-COO), 1.98 (dt, 2H, NH-CH_2_-C*H*_2_-CH_2_).

### 2.3 Iodine comonomer/reference synthesis

To enable visualization of the polymers with CECT and gain control over their net charge, we synthesized two iodinated comonomers with net charge of either +2 (cationic iodinated comonomer (CATIC) or −2 (anionic iodinated comonomer (ANIC), Fig. 1B). As the comonomers were derived from 3-amino-2,4,6-triiodoisophthtalic acid (ATIIPA), they both contain three iodine atoms per molecule and are structurally similar, thus ensuring comparability. CA2+, another derivative of ATIIPA, was also synthesized and used as a reference contrast agent in our experiments [17].

#### 2.3.1 ANIC monomer synthesis

1.0 g of 3-amino-2,4,6-triiodoisophthtalic acid (1.79 mmol) was dissolved in 10 mL of acetonitrile to which 2.5 μL of sulfuric acid (4.5 mg, 44 μmol, 0.025 eq.) was added and the solution cooled to 4 °C. 824 μL of methacrylic anhydride (852 mg, 5.37 mmol, 3.0 eq.) was added dropwise over 30 min and the reaction mixture stirred overnight (Fig. S1B). The mixture was concentrated under reduced pressure to 5 mL, triggering precipitation, and cooled to −20 °C. After washing with cold acetonitrile, the target compound was isolated as a light brown solid (674 mg, 1.08 mmol, 60 %). ^1^H NMR (400 MHz, D_2_O): *δ* (ppm) 10.00 (s, 1H, N*H*-COCCH_3_=CH_2_), 5.93 (m, 1H, NH-COCCH_3_=CH*H*), 5.57 (m, 1H, NH-COCCH_3_=C*H*H), 1.96 (m, 3H, N*H*-COCC*H*_3_=CH_2_).

#### 2.3.2 CA2+ synthesis

CA2+ was synthesized following the previously established protocol by Joshi et al. [17] (Fig. S1D).

##### 2.3.2.1 5-Amino-2,4,6-triiodoisophthaloyl dichloride (1)

3-amino-2,4,6-triiodoisophthalic acid (18.5 g, 33.1 mmol) was placed in 75 mL thionyl chloride and refluxed for 8 h. Completion of the reaction was confirmed via TLC through appearance of a single, more apolar, product. The reaction mixture was concentrated under reduced pressure, dissolved in ethyl acetate and washed three times with 1:1 sat. NaHCO_3_/brine. The combined organic layers were dried over NaSO_4_ and the solvent removed under reduced pressure to yield a yellow solid which was used directly for subsequent reactions without further purification or characterization.

##### 2.3.2.2 5-Aminoacyl-2,4,6-triiodisophthaloyl chloride (2)

Crude 1 (3.0 g, 5.0 mmol) was dissolved in 8 mL of anhydrous DMA and stirred on ice. Acetyl chloride (1.1 mL, 15 mmol, 3 eq.) was added dropwise over 1 h, and the reaction stirred overnight. TLC indicated complete conversion by appearance of a more polar product. The reaction mixture was concentrated to ∼2–3 mL under reduced pressure, diluted with 1 mL of methanol and precipitated in 300 mL of 1:1 ethyl acetate/hexane. After centrifugation (4300×*g*, 10 min), the crude pellet was dried under reduced pressure to yield a light brown, crystalline product that was used directly for the subsequent reaction without further purification or characterization.

##### 2.3.2.3 (Boc-ethylenediamine)-5-aminoacyl-2,4,6-triiodoisophtaloyl bis-amide (3)

Crude 2 (2.13 g, 3.3 mmol) was dissolved in 8 mL of anhydrous DMA with 1.37 mL of triethylamine (10.0 mmol, 3 eq.). 1.0 mL of *N-*boc-ethylenediamine (7.0 mmol, 2.1 eq.) diluted with 1.5 mL of anhydrous DMA was added dropwise over 1 h and the reaction mixture stirred at room temperature overnight. Full conversion was assessed with TLC through the appearance of an apolar product. The mixture was concentrated under reduced pressure to ∼2–3 mL, precipitated in 150 mL of 1:1 ethyl acetate/hexane, and centrifuged (4300×*g*, 10 min). The crude pellet was dried under reduced pressure to yield a light brown solid that was used directly for the subsequent reaction without further purification or characterization.

##### 2.3.2.4 (Ethylenediamine)-5-aminoacyl-2,4,6-triiodoisophthaloyl amide, trifluoroacetate salt (CA2+)

Crude 3 (500 mg, 0.56 mmol) was dissolved in trifluoroacetic acid with 4 v/v% triisopropylsilane and 4 v/v% dH2O and shaken on an orbital shaker for 3 h. The reaction mixture was concentrated to ∼1 mL under reduced pressure and precipitated in 20 mL cold diethyl ether. After sitting on ice for 20 min and centrifugation (4300×*g*, 4 °C, 10 min), the pellet was dried under reduced pressure to yield CA2+ as a pale yellow solid with a combined yield of 40 % across all steps. ^1^H NMR (400 MHz, DMSO-*d*_6_): *δ* 10.00 (s, 1H, N*H*-COCH_3_), 8.81 (m, 2H, CON*H*-CH_2_-CH_2_-NH_3_ x 2), 7.95 (s, br, 6H, CH_2_-N*H*_3_ x 2), 3.46 (m, 4H, CONH-C*H*_2_-CH_2_-NH_3_ x 2), 2.99 (m, 4H, CONH-CH_2_-C*H*_2_-NH_3_ x 2), 2.04 (s, 3H, NH-COC*H*_3_).

#### 2.3.3 CATIC monomer synthesis

The CATIC monomer was synthesized analogously as CA2+, simply replacing the acetyl chloride from 2.4.2 with acryloyl chloride (Fig. S1C).

##### 2.3.3.1 5-Acrylamido-2,4,6-triiodoisophthaloyl dichloride (4)

Crude 1 (3.0 g, 5.0 mmol) was dissolved in 8 mL of anhydrous DMA and stirred on ice. Acryloyl chloride (2.5 mL, 30 mmol, 6 eq.) was added dropwise over 1 h and the reaction stirred overnight. TLC indicated complete conversion by appearance of a more polar product. The reaction mixture was concentrated to ∼2–3 mL under reduced pressure, diluted with 1 mL of methanol and precipitated in 300 mL of 1:1 ethyl acetate/hexane. After centrifugation (4300×*g*, 10 min), the crude pellet was dried under reduced pressure to yield a light brown, crystalline product that was used directly for the subsequent reaction without further purification or characterization.

##### 2.3.3.2 (Boc-ethylenediamine)-5-aminoacryl-2-4,6- triiodoisophthaloyl bis-amide (5)

Crude 4 (2.20 g, 3.4 mmol) was dissolved in 8 mL of anhydrous DMA with 1.44 mL of triethylamine (10.2 mmol, 3 eq.). 1.1 mL of *N-*boc-ethylenediamine (7.1 mmol, 2.1 eq.) diluted with 1.5 mL of anhydrous DMA was added dropwise over 1 h and the reaction mixture stirred at room temperature overnight. Full conversion was assessed with TLC through appearance of an apolar product. The mixture was concentrated under reduced pressure to ∼2–3 mL, precipitated in 150 mL of 1:1 ethyl acetate/hexane, and centrifuged (4300×*g*, 10 min). The crude pellet was dried under reduced pressure to yield a light brown solid that was used directly for the subsequent reaction without further purification or characterization.

##### 2.3.3.3 (Ethylenediamine)-5-aminoacryl-2-4,6- triiodoisophthaloyl amide, trifluoroacetate salt (CATIC)

Crude 5 (500 mg, 0.56 mmol) was dissolved in trifluoroacetic acid with 4 v/v% triisopropylsilane and 4 v/v% dH2O and shaken on an orbital shaker for 3 h. The reaction mixture was concentrated to ∼1 mL under reduced pressure and precipitated in 20 mL cold diethyl ether. After sitting on ice for 20 min and centrifugation (4300×*g*, 4 °C, 10 min), the pellet was dried under reduced pressure to yield CATIC as a pale yellow solid with a combined yield across all steps of 48 %. ^1^H NMR (400 MHz, DMSO-*d*_6_): *δ* 10.20 (s, 1H, N*H*-COCH=CH_2_), 8.81 (m, 2H, CON*H*-CH_2_-CH_2_-NH_3_ x 2), 7.94 (s, br, 6H, CH_2_-N*H*_3_ x 2), 6.46 (dd, 1H, COC*H*=CH_2_), 6.26 (dd, 1H, COCH=C*H*H), 5.82 (dd, 1H, COCH=CH*H*), 3.46 (m, 4H, CONH-C*H*_2_-CH_2_-NH_3_ x 2), 2.99 (m, 4H, CONH-CH_2_-C*H*_2_-NH_3_ x 2).

### 2.4 RAFT polymerization

All polymers in this study were synthesized following the below protocol with the co-monomer ratios adjusted according to Table 1:

**Table 1:** **Characterization of pCBAA polymers with different ratios of ANIC/CATIC comonomer:** Mw: weight average molecular weight, Mn: number average molecular weight, PDI: Polydispersity index, Rh: hydrodynamic radius. N = 3.

Sample	ANIC [mol%]	CATIC [mol%]	Expected Iodine [wt%]	Measured Iodine [wt%]	Mw [kDa]	Mn [kDa]	PDI	Rh [nm]	Zeta pot [mV]
A20	20	–	24.7	27.0 ± 1.9	67.5 ± 1.1	48.3 ± 1.0	1.40 ± 0.01	4.30 ± 0.09	−39.6 ± 2.2
A10	10	–	14.2	20.2 ± 1.4	47.7 ± 1.5	44.4 ± 0.8	1.08 ± 0.02	3.71 ± 0.05	−27.8 ± 0.7
A5	5	–	7.7	10.6 ± 2.6	38.1 ± 0.3	35.5 ± 0.1	1.07 ± 0.01	3.45 ± 0.03	22.9 ± 0.9
N20	10	10	24.2	21.1 ± 3.7	49.8 ± 0.7	46.2 ± 0.6	1.08 ± 0.00	3.66 ± 0.12	24.0 ± 0.9
N10	5	5	14.0	10.2 ± 0.3	42.1 ± 0.7	39.8 ± 0.9	1.06 ± 0.01	3.51 ± 0.04	25.7 ± 0.3
N5	2.5	2.5	7.6	5.7 ± 0.3	39.2 ± 2.4	36.8 ± 2.1	1.07 ± 0.00	3.5 ± 0.13	28.1 ± 1.5
C40	–	40	36.6	18.1 ± 1.4	51.5 ± 3.5	45.5 ± 2.2	1.13 ± 0.02	2.51 ± 0.57	36.8 ± 0.4
C20	–	20	23.6	10.8 ± 1.2	32.4 ± 1.7	30.4 ± 1.3	1.07 ± 0.01	2.98 ± 0.10	36.0 ± 0.7
C10	–	10	13.8	6.4 ± 0.3	32.4 ± 7.0	27.8 ± 2.6	1.16 ± 0.14	3.21 ± 0.17	26.3 ± 0.9
CA2+	–	–	41.7	32.1 ± 2.1	0.69	0.69	1	–	25.6 ± 3.7
Iohexol	–	–	46.4	–	0.82	0.82	1	–	1.93 ± 0.13

390.2 mg of CBAA (1708 μmol, 95 mol%), 56.4 mg of ANIC (90 μmol, 5 mol%), 0.59 mg of acryloxyethyl thiocarbamoyl rhodamine B (0.90 μmol, 0.05 mol%), 4.8 mg of cyanomethyl dodecyl trithiocarbonate (15 μmol, 2.1 mol%) and 4.5 mg of V-65 (17.9 μmol, 2.5 mol%) were dissolved in 2 mL of trifluoroethanol. After purging with N2 for 10 min, the solution was stirred at 55 °C overnight. The reaction mixture was diluted with dH2O, transferred to regenerated cellulose dialysis membranes (3.4 kDa MWCO, Spectrum Laboratories, NZL), dialyzed against dH2O (3 × 12h) and lyophilized to yield the purified polymer as a pink, fluffy solid (290 mg, 63 % yield).

### 2.5 Determination of iodination degree

Polymer solutions were prepared in triplicates at 0.5 mg/mL in 1x PBS and their photo absorbance read at 240 nm using a NanoDrop OneC device (Thermo Fisher Scientific, USA). The degree of iodination was then determined using a standard curve of ATIIPA in 1x PBS.

### 2.6 Size-exclusion chromatography

Polymers were dissolved in aq. 0.02 w/v% NaN_3_ and 0.1 M NaNO_3_ at a concentration of 1 mg/mL and their size determined with a high-performance size-exclusion chromatography device (OMNISEC, Malvern Panalytical, USA) equipped with two A′6000M columns in series (Viscotek, 8.0 × 300 mm), an LALS/RALS detector, an RI detector, a UV detector and a viscosimeter (all Malvern Panalytical, UK). Samples were measured in triplicates at a column temperature of 30 °C and 0.7 mL/min, and the analysis was performed using the OMNISEC software version v.10.30 (Malvern Panalytical, UK) using narrow molecular weight distribution polyethyleneoxide (24 kDa) as the standard.

### 2.7 Zeta potential

Polymer solutions were prepared at 10 mg/mL in dH_2_O and the zeta potential was measured on a Zetasizer Nano device (Malvern Panalytical, UK). Every sample was measured three times with at least 10 reads per measurement.

### 2.8 Bovine osteochondral plug harvest and culture

4.6 mm diameter bovine osteochondral plugs with cartilage thickness of 1.0 ± 0.1 mm were harvested from the femoral condyles of freshly slaughtered 1-to-2-year-old cows (Angst AG, CH) using soft delivery system™ tools (Sulzer Medica, CH). After washing in sterile 1x PBS supplemented with 1x Antibiotic-Antimycotic (Thermo Fisher Scientific, USA) for 2 h with several changes of the wash solution, the plugs were placed in silicone fixtures (SYLGARD 184, Dow Chemical, USA) in the bottom of 6-well plates with only the cartilage surface exposed. To allow the tissue to recover from the harvesting procedure, the disks were kept in culture medium (DMEM + GlutaMAX (high glucose, pyruvate, Thermo Fisher Scientific, USA) with 1x ITS (Thermo Fisher Scientific, USA) and 10 μg/mL Gentamycin) under standard cell culture conditions (37 °C, 5 % CO2, 95 % humidity) for at least 4 days before treatment with 10 ng/mL human IL-1β (PeproTech, UK) for up to 3 weeks. At the different timepoints, the samples were fixed with 4 % formaldehyde for 2 h and stored in 1x PBS at 4 °C until further use. Note that the data was collected from 5 to 2 different donors for Fig. 2, Fig. 3, respectively.

**Fig. 2:**
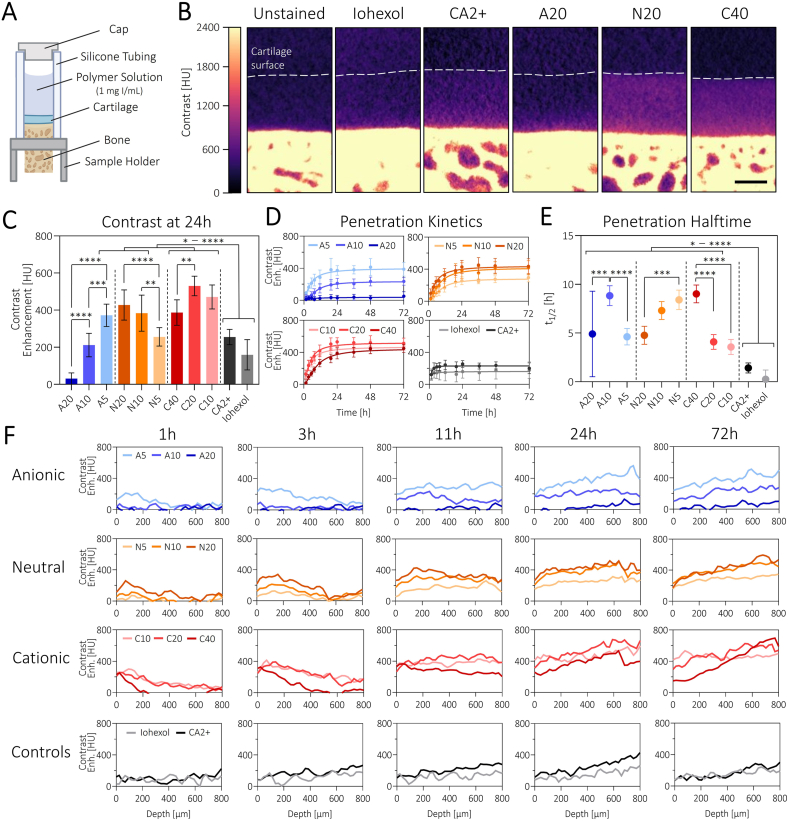
Polymer contrast agents show charge-dependent uptake into healthy cartilage: A) Schematic illustration of the experimental setup. B) CECT cross sections of osteochondral plugs after incubation with contrast agents for 24 h in comparison to the unstained control. Scale bar: 500 μm. C) Averaged contrast enhancement over the whole cartilage volume indicates increased, charge-dependent contrast for most polymers compared to the reference samples CA2+ and iohexol. D) Penetration kinetics are also charge-dependent and reveal (E) increasing and decreasing penetration halftimes for increasing iodine loading for C and N polymers, respectively. F) As the polymers penetrate the cartilage tissue, their zonal distribution changes over time, finally reaching an equilibrium with higher concentration in the deeper zones compared to the surface, to match the local GAG concentrations. This is particularly striking for the most cationic polymer C40. N = 8. Figure created with Biorender.

**Fig. 3:**
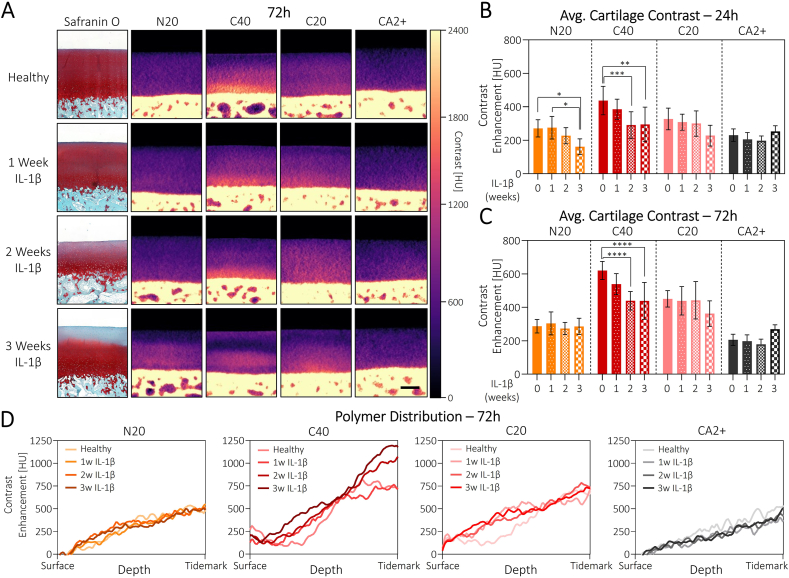
**Polymer contrast agents detect different stages of cartilage degradation:** A) Histological images of safranin O-stained cartilage tissue at different stages of degradation (left) in comparison to the respective CECT micrographs after 72 h of polymer incubation (right). Scale bar: 500 μm. B) Analysis of bulk averaged contrast enhancement after 24 h of polymer incubation reveals diagnostic potential of N20 and C40. C) After 72 h, the differences in N20 contrast are lost due to an unidentified mechanism, whereas the ones for C40 become more pronounced. D) Normalized polymer distribution profiles after 72 h show decreased surface staining for C40 and C20 polymers with increasing tissue degradation. The general trend for increasing contrast with increasing depth, however, remains visible for all contrast agents and tissue states. Penetration profiles are plotted in relative scale to accommodate differences in cartilage thickness between the different samples. N = 8.

### 2.9 X-ray micro-computed tomography

For a 1:1 comparison between contrast agent and histological staining, a 1 mm full-thickness biopsy (KAI MEDICAL, JPN) was taken from the cartilage of each plug prior to starting incubation with the contrast agents and processed for histology. The plugs were fixed in 4 mm silicone tubing into which 200 μL of a 1 mg I/mL contrast agent solution in 0.9 w/v% NaCl was added. The tubing was sealed with a cap and each sample was scanned once per timepoint with a μCT 45 device (Scanco Medical, CH) in custom-made holders at 100 % humidity. The scans were acquired at 55 kV, 145 μA and 8W using an 0.5 mm aluminum filter (1500 projections/180°, 1.9 s integration time per projection, 14.6 μm voxel size, 47.5 min per sample). Pixel intensities were recorded from the cartilage surface to the tidemark at 8 different sample locations and the bulk contrast enhancement calculated by averaging all the pixel values and subtracting the background contrast of an untreated sample. The penetration halftime for the different sample solutions was calculated by applying a sigmoidal fit to the curves with the penetration kinetics and extracting the IC50 value. The partition coefficient (i.e. the ratio of intra- and extra-cartilage polymer concentration) was calculated by dividing the contrast enhancement within the cartilage tissue by the contrast enhancement of the applied solution (after subtraction of the saline baseline contrast).

### 2.10 Histology

Samples were dehydrated in several steps to 70 % ethanol, followed by automated paraffinization on a Milestone Logos J device (Milestone, ITA). 5 μm-thick longitudinal sections were prepared with a microtome (HM 325, Microm, GER). After rehydration, the tissue sections were incubated with Weigert's iron hematoxylin for 5 min, washed in dH2O (3 × 30s), submerged in 1 % acid alcohol (2s), washed in dH2O (3 × 30s), stained in 0.02 % fast green (1 min), destained in 1 % acetic acid (30s) and stained in 0.1 % safranin O (30 min). After washing in 95 % ethanol (3 × 30s), dehydration in 100 % ethanol (2 × 1 min), clearing in xylene (2 × 1 min) and air-drying, the sections were coverslipped using Eukitt mounting medium and imaged on a Pannoramic 250 Flash II slide scanner (3DHistech, HUN). For the correlation analysis, the safranin O staining intensity was measured across the full cartilage thickness after color deconvolution in Fiji ImageJ v1.51n. Concretely, pixel values were converted to intensity ($Intensity=\log\;\left(\frac{255}{Pixel\;value}\right)$) and then correlated 1:1 with the calculated contrast enhancement from the same area. Correlation was assessed by calculating the corresponding Pearson's correlation coefficient R^2^. P values for all correlation pairs in our study were <0.0001.

### 2.11 Statistical analysis

All statistical analysis was performed with GraphPad Prism v. 10.1.2 (GraphPad, USA). One-way analysis of variance (ANOVA) was performed with a Tukey's multiple comparisons test for the data shown in all figures. The number of replicates was 8. To minimize the effect of donor-to-donor variability, the penetration experiments were performed with samples from 5 different donors that were distributed evenly between the conditions. Statistical significance is indicated with asterisks: ∗p < 0.05, ∗∗p < 0.01, ∗∗∗p < 0.001, ∗∗∗∗p < 0.0001.

## 3 Results and discussion

### 3.1 Polymer synthesis and characterization

For the anionic iodinated comonomer (ANIC), we adapted a previously established protocol from *An Le* et al. to modify the amine of ATIIPA with methacrylic anhydride [25]. For the cationic iodinated comonomer (CATIC), we took inspiration from the study by *Joshi* et al.*,* which first described the contrast agent CA4+ and compared it to the structurally related molecules CA2+ and CA1+ [17]. Though CA2+ was found to be inferior to CA4+ in terms of contrast enhancement, its structure is very similar to ANIC with the two anionic carboxylic acid moieties each being coupled to an ethylenediamine group, thus giving the molecule an overall positive charge. By replacing the acetyl group with an acrylamide group, we adapted CA2+ to serve as our polymerizable CATIC.

With these two iodinated comonomers in hand, CBAA copolymers of three different classes were synthesized: Cationic (C, n_CATIC_ > 0, n_ANIC_ = 0), neutral (N, n_CATIC_ = n_ANIC_ > 0) and anionic (A, n_CATIC_ = 0, n_ANIC_ > 0). To evaluate the effect of the iodination degree, several copolymers with different ratios of iodinated comonomer(s) to CBAA were synthesized for each of the three classes. Each polymer of the resulting library is denoted by a letter to signify the class and a number to indicate the molar fraction of iodinated comonomer(s) in the polymerization feed, i.e. “N10” describes a CBAA copolymer with 5 mol% of each ANIC and CATIC. To ensure comparable polymer lengths across our library, all copolymers were synthesized through reversible-addition-fragmentation-transfer (RAFT) polymerization with a target mass of around 40 kDa.

Due to the absence of spectrometrically isolated protons for ANIC once incorporated into the polymer, it is not possible to determine the exact comonomer loadings in the purified polymers. Nevertheless, the trend in iodine content indicates that CATIC copolymerized a little less efficiently compared to ANIC, particularly at high comonomer fractions (Table 1): Whereas A20 had a measured iodine content of 27.0 ± 1.9 wt% in range of the expected value of 24.7 wt%, the values for N20 and particularly C20 were reduced with 21.1 ± 3.7 wt% and 10.8 ± 1.2 wt%, respectively (Table 1). To compensate for this, a C40 polymer was prepared, which finally reached a comparable degree of iodination of 18.1 ± 1.4 wt%. Characterization with multi-angle light scattering revealed generally comparable molecular weights for all polymers between 30 and 70 kDa (Table 1). Generally, the molecular weight was increased for increasing iodine content, which is in accordance with the higher molecular weight of the iodinated co-monomers compared to CBAA. The hydrodynamic radii were also comparable and well below the cartilage pore size of 6 nm, thus eliminating any steric limitations regarding cartilage penetration [26].

Finally, all polymers were characterized with respect to their charge, employing zeta potential measurements. For the A polymers, we observed the expected trend with an increasing negative charge with increased ANIC loading (Table 1). Though A20 and A10 had a strongly negative zeta potential, the value for A5 was positive, indicating that the 5 mol% ANIC were not sufficient to compensate for the base-positive charge of the unmodified pCBAA. In one previous study, the cationic charge density on pCBAA polymers was estimated at around 10 mol% and therefore in a similar range to the anionic charge density introduced through copolymerization with ANIC [23]. However, the strongly positive zeta potential of A5 suggests that some of the ANIC carboxylic acids might also become protonated once incorporated into the polymer, as the charge imbalance was bigger than expected. One could imagine a similar intramolecular mechanism to that in unmodified pCBAA, where the highly crowded environment significantly raises the pKa of the carboxylic acid groups. For the N polymers, all samples showed a zeta potential in a similar range to unmodified pCBAA which is slightly positive, indicating that the introduction of the iodinated comonomers did not significantly influence the overall charge [23,27]. The zeta potential did slightly decrease with higher comonomer loading, which can be explained by the decreased ability of CATIC to be polymerized compared to ANIC. Finally, the C polymers all displayed positive values, with C20 and C40 having a more positive zeta potential compared to C10. Unexpectedly, the difference between C20 and C40 was negligible, and it seems the zeta potential reaches a plateau above a certain CATIC loading. Though the exact mechanism remains unclear, it seems likely that this is a consequence of the increasing intrapolymer interactions of the amines on CATIC. Previous studies on polyamines have found that their pKa was decreased when the intramolecular loading was increased, thereby leading to a decreased positive charge at physiological pH [[28], [29], [30]].

### 3.2 Charge-sensitive cartilage penetration kinetics into healthy cartilage

To evaluate the potential of our polymers as contrast agents, we first incubated them with healthy, untreated osteochondral plugs harvested from adult bovine knee joints. To ensure unilateral penetration, the plugs were inserted into silicone tubing and the polymer solution was applied onto the cartilage surface (Fig. 2A). All sample solutions were prepared at an iodine concentration of 1 mg I/mL, including the two reference contrast agents CA2+ and iohexol. The osmolarities of the solutions did not show any significant differences and were considered negligible (Table S1). After 24 h of incubation, all contrast agents enabled visualization of the border between the cartilage tissue and the sample solution by CECT, with iohexol and the A20 polymer being the only two exceptions (Fig. 2B). In addition to visualizing the cartilage-solution boundary, the bulk contrast enhancement averaged over the whole cartilage tissue volume was furthermore significantly increased compared to the cationic reference CA2+ for most candidates (Fig. 2C). This improvement is most likely due to the greatly increased net charge of the polymers compared to the small molecule contrast agent which allows for a greater driving force into the tissue as well as a stronger binding due to multivalency [31]. When comparing candidates within one polymer class, the A polymers showed the expected, charge-correlated increase in contrast enhancement with decreasing comonomer loading (Fig. 2C). Despite comparable zeta potentials, N5 only reached 60 % of the contrast enhancement achieved by N20, with N10 in the middle of the two. This can possibly be explained by the relatively high polymer concentration of the N5 sample, which was at 17.4 mg/mL and therefore in a range where the cartilage tissue starts to get saturated and the partition coefficient is decreased compared to lower concentrations [23]. For the C polymers there was no trend, with C20 performing better than both C10 and C40. Similar observations were also made with respect to the partition coefficients which were at around 2–3 for most of the polymers and generally followed the same charge-dependent trends as for the contrast enhancement (Fig. S2). With a partition coefficient of 1.1 and 0.5 for CA2+ and iohexol, respectively, our reference contrast agents showed very poor cartilage specificity compared to our multivalent polymers. Nevertheless, a partition coefficient of 2–3 is still relatively low in comparison to other cartilage targeting agents which we attribute to the high polymer concentrations used in these experiments, thereby quickly saturating the cartilage tissue [14,32].

While the difference in bulk contrast enhancement between C10 and C20 can most certainly be explained by a decreased electrostatic driving force, the difference for C40 is mostly a consequence of slower penetration kinetics (Fig. 2D/E). Though almost all polymers reached equilibrium after 24 h (i.e. the point at which no further change in attenuation was observed), C40 continued to penetrate and reached similar a contrast enhancement to C10 and C20 after 72 h. With a penetration halftime of 9.0 ± 0.9h, C40 diffused significantly more slowly into the cartilage tissue compared to C20 and C10, with 4.1 ± 0.8h and 3.6 ± 0.8h, respectively. This matches the findings of Bajpayee and coworkers: that there exists a threshold beyond which the addition of more positive charges starts to restrict tissue penetration of cationic macromolecules due to their increasingly strong interactions with the anionic cartilage ECM [15,33]. Though the zeta potential measurements do not indicate an increased net charge on C40 compared to C20, these measurements were conducted in a highly controlled environment, and it is likely that the local chemical environment at the cartilage surface would also influence the degree of protonation and thus the overall charge.

For the N polymers, the polymers that had enabled increased contrast enhancement also showed faster penetration kinetics. This was somewhat unexpected as – assuming identical charge and size of all N polymers – the increased concentration gradient for N5 compared to N20 should increase its equilibration speed, following Donnan theory [34,35]. Further characterization will be necessary to fully understand the penetration trends of the N polymers. While the A polymers did not display a trend for penetration halftime, the most significant differences in the dataset were found when comparing our polymers to the small molecule reference contrast agents. With penetration halftimes of 1.4 ± 0.5h and 0.3 ± 1.0h, both CA2+ and iohexol expectedly showed extremely fast penetration compared to all the investigated polymers. Though the determined polymer penetration kinetics are certainly not ideal for a clinical setting, where penetration should occur as quickly as possible, the achieved contrast levels at equilibrium should remain superior, as an increased molecular weight will similarly enable a longer residence time within the joint capsule, thereby increasing the chance of cartilage uptake.

Finally, we also investigated the local polymer distribution within the cartilage tissue throughout the penetration process and found the expected trend from mainly surface localization at the early timepoints to a more even distribution at the later timepoints (Fig. 2F). At equilibrium, all polymers had, in fact, a higher local concentration in the deeper zones of the tissue compared to the surface, which matches the zonal GAG distribution. For the C40 polymer, this trend was particularly pronounced with an average contrast enhancement of 151 ± 3 HU in the top 100 μm and 663 ± 42 HU at the bottom 100 μm of the tissue. As previous studies determined the average GAG concentration at around 30 and 110 mg/mL for the superficial and deep zone respectively, the C40 polymer reproduced these quantitative differences the most accurately [3,4]. While being the most GAG-sensitive, the depth analysis also showed again that the C40 polymer takes relatively long to reach equilibrium and that this polymer displayed the highest levels of surface localization during the early timepoints of this experiment. We would expect that in a more dynamic environment (i.e. shaking as well as applying compression and shear), the penetration kinetics would increase. However, further experiments to confirm this hypothesis need to be conducted to better predict *in vivo* behavior. Therefore, we selected not only C40 but also C20 and N20 as well as the CA2+ control for the subsequent experiment.

### 3.3 Polymer contrast agents detect local cartilage degradation

To investigate whether our polymer contrast agents could distinguish between healthy and degraded cartilage tissue, we cultured osteochondral plugs in the presence of the inflammatory cytokine interleukin-1β (IL-1β). Treatment with IL-1β is one of the most widely used methods to create *in vitro* models of OA, as it triggers a variety of catabolic biochemical pathways leading to a degraded collagen network, loss of GAGs, and inferior mechanical properties. To create tissues with different degrees of degradation, the plugs were treated with IL-1β for either 1, 2 or 3 weeks. While 1 week of inflammatory treatment did not induce any noticeable degradation in the safranin O staining, we observed a loss of superficial GAGs after 2 weeks, which was even more pronounced at week 3 (Fig. 3A). Analysis of the bulk contrast enhancement after 24 h of polymer incubation revealed that both N20 and C40 were able to distinguish between the different degradation states (Fig. 3B). While not all comparisons between the groups reached statistical significance, there is a strong trend towards a decreased bulk contrast enhancement for longer IL-1β exposure for these two polymers. Despite not reaching statistical significance, the same – albeit weaker – trend was also observed for C20. We attribute this statistical insignificance to the minimal GAG loss which results in only a minor charge difference, making it undetectable for C20.

For CA2+, on the other hand, there was no trend, with the 3-week IL-1β group reporting the highest bulk contrast enhancement of all tissue states. After 72 h, the trends for the cationic polymers remained the same and even became stronger in the case of C40 (Fig. 3C). For the N20 polymer, however, the trend was now no longer visible, with all tissues being equally stained. We can currently only hypothesize as to why this was the case, since the penetration kinetics for N20 indicate complete equilibration after 24 h, with only very minor local changes beyond this timepoint (Fig. 2F). One possible explanation is that with a decreased GAG content upon IL-1β stimulation, the electrostatic driving force into the tissue was decreased, therefore requiring more time to reach full equilibration. Nevertheless, the equilibrated state itself should also be influenced by the local charge densities. Moreover, this should also apply analogously to C40 and C20, which is not the case, thereby suggesting the involvement of an additional mechanism. Depending on the application, it might, however, not be necessary or even possible to wait for full equilibration (i.e., in a clinical setting), in which case N20 might still be a viable candidate.

Beyond the differences in bulk contrast enhancement, a closer look at the corresponding micrographs after 72 h of incubation also indicated striking differences regarding the local polymer distribution within the different tissues (Fig. 3A). While for the healthy samples we again observed a continuous increase in contrast for increasing depth, IL-1β treated samples showed a more discontinuous profile, which matched the trends in the safranin O stainings (Fig. 3D). Especially for the C40 sample, the inflamed tissues displayed a region of disproportionately low contrast below the surface, which became more extended the longer the samples had been exposed to IL-1β. At the surface itself, however, the contrast for C40 was actually greater for the 3w IL-1β tissue compared to the others (Fig. 3A/D). Though 72 h was found to be sufficient to reach the equilibrium for the C40 sample in healthy cartilage, the reduced amount of anionic GAGs in the inflamed tissues is expected to reduce the electrostatic driving force into the cartilage, thereby requiring even longer incubation times for the slowest-penetrating C40 polymer. Finally, the C40 contrast immediately above the tidemark was also substantially lower for the 2w and 3w IL-1β samples compared to the healthy group – a feature that was not visible in the histology images and might indicate superior sensitivity of our polymers in the high GAG concentration range compared to the non-quantitative Safranin O staining. Similar to the trends in bulk contrast enhancement, the C20 polymer also showed potential to visualize the GAG-depleted surface layer in the 3w IL-1β. The other two inflamed groups were, however, indistinguishable from the healthy sample. Furthermore, there was no local sensitivity for either N20 or CA2+, which only detected the general zonal trend towards increased GAGs in the deeper zones, with no differences between the tissue states.

**Fig. 4:**
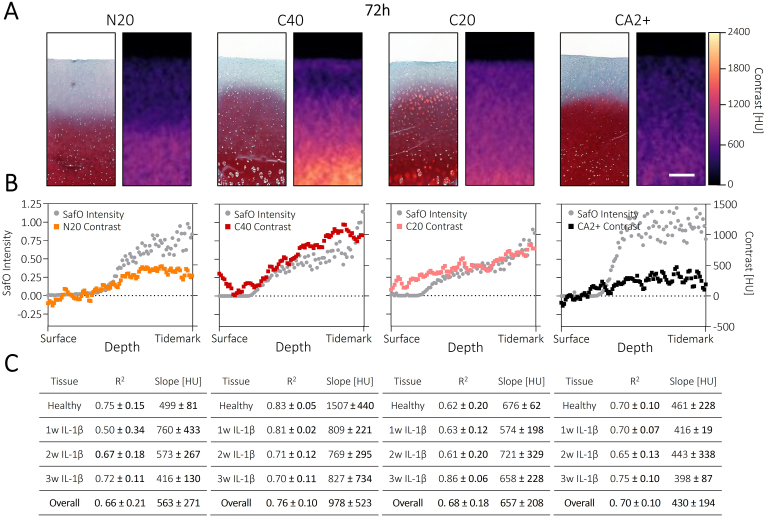
**C40 polymer shows best correlation with safranin O staining:** A) Side-by-side comparison of safranin O and polymer staining of the same cartilage sample. Scale bar: 250 μm. B) Overlay of local safranin O intensity profiles with CECT results for the plugs shown in panel A. Penetration profiles are plotted in relative scale to accommodate differences in cartilage thickness between the different samples. C) Averaged correlation results across the replicates within one tissue state and across all tissue states for the investigated contrast agents, indicating C40 as the most locally specific and sensitive contrast agent. N = 4.

To investigate the potential of our method as a non-destructive, 3D histology substitute in more detail, we also calculated the sample-specific correlation coefficients between the local safranin O staining intensity and contrast enhancement (Fig. 4A). This revealed moderate correlation coefficients >0.65 for all the investigated contrast agents across all disease states (Fig. 4B). Despite not being able to detect the transition between GAG-depleted and intact regions, CA2+ still achieved relatively good correlation, as it reproduced the general GAG increase towards the deeper layers. More often than not, N20 and C20 were also able to visualize the border of GAG depletion (Fig. 4A); however, this did not significantly increase the correlation coefficient compared to CA2+. As the level of GAG depletion was relatively mild in all our models, it seems the correlation coefficients of these moderately specific contrast agents were mainly driven by the general trend in GAG concentration with depth. Still, the C40 polymer that visualized the GAG border most strongly and accurately achieved an increased correlation coefficient of 0.76 ± 0.10, supporting it as the most locally specific of the investigated contrast agents. C40 also showed the highest correlation slope, indicating the highest sensitivity. The only drawback for C40 was that, as described above, there was often a thin layer of polymer at the surface of the degraded tissues due to incomplete equilibration. In addition to longer incubation times, this issue could, however, also be solved by the introduction of a washing step before imaging, to remove the surface layer and enable a further improved correlation with the local GAG distribution.

## 4 Conclusion

In this study, we introduced a new class of polymeric, iodine-bearing contrast agents based on zwitterionic carboxybetaine acrylamide. By combining the concepts of modularity and multivalency, we were able to control the overall charge and iodine content of the contrast agents. While the most strongly anionic A20 polymer showed no cartilage uptake whatsoever, N and C polymers enabled moderate-to-good contrast enhancement and in some cases reached a more than two-fold improvement compared to the established small molecule contrast agents iohexol and CA2+, thereby supporting their improved cartilage affinity. While CA2+ was only able to detect the general trend of increasing GAGs with increasing tissue depth, C20 and particularly C40 could also discriminate between healthy and degraded samples, both with regard to the bulk contrast enhancement and to local GAG depletion within the specific tissues.

Despite C20 and C40 showing superior results compared to CA2+ for the evaluation of the cartilage tissue state, their reduced penetration speed remains a major limitation. Though the differences in bulk contrast enhancement may already be visible within a few hours, the local differences will only be trustworthy after full equilibration is reached. In that regard, C40 might not be the most suitable, as it was found to have a tendency to get stuck at the cartilage surface, therefore requiring very long equilibration times, particularly for tissues with reduced GAG content. This could theoretically be addressed by using C20 instead and further reducing the molecular weight, though this would most certainly also compromise its specificity to some extent. Another limitation is the rather low iodine loading of our polymers compared to CA2+ and iohexol. Despite achieving a significantly stronger contrast at 1 mg I/mL, the heavier polymers would most likely reach cartilage saturation already at lower iodine concentrations than CA2+, thereby restricting the maximum contrast that can be achieved. Though a CATIC homopolymer would greatly increase the iodine loading, it would most certainly suffer from even worse penetration kinetics than C40. The use of polymers based on a zwitterionic iodinated monomer together with a small mole-fraction of CATIC to ensure a net-positive charge would therefore be the optimal solution and represents a promising direction for future studies.

In summary, our study shows the importance of multivalency and charge control for the accurate visualization of GAGs in cartilage tissue via CECT and presents zwitterionic polymers as potential candidates for this. By enabling a nondestructive, 3D visualization of the GAG distribution in cartilage specimen, CECT represents a powerful tool for OA research that could easily be included in the routine analysis pipeline of preclinical studies, which often already includes CT scanning for the evaluation of bone parameters. Moreover, a combination with other complementary contrast agents such as phosphotungstic acid or labelled collagen-binding peptides with affinity towards collagen II [[36], [37], [38]] might enable visualization of several ECM components simultaneously via spectral computed tomography [39]. With regard to a potential application *in vivo*, the high specificity and therefore low required dose represent a promising starting point for these materials. However, studying the influence of movement on the penetration kinetics (i.e. shaking as well as applying compression and shear) and further biocompatibility studies will be required, to fully assess their translational potential.

## CRediT authorship contribution statement

**Patrick Weber:** Writing – review & editing, Writing – original draft, Visualization, Validation, Methodology, Formal analysis, Data curation, Conceptualization. **Annalena Maier:** Writing – review & editing, Writing – original draft, Visualization, Validation, Methodology, Formal analysis, Data curation, Conceptualization. **David Fercher:** Writing – review & editing, Writing – original draft, Visualization, Validation, Supervision, Methodology, Formal analysis, Conceptualization. **Maryam Asadikorayem:** Writing – review & editing, Methodology, Investigation. **Marcy Zenobi-Wong:** Writing – review & editing, Supervision, Funding acquisition.

## Data availability statement

P. Weber, A. Maier, D. Fercher, M. Asadikorayem, M. Zenobi-Wong, https://doi.org/10.3929/ethz-b-000681325, ETH Zurich Research Collection 2024.

## Funding

This work was supported by the 10.13039/100000001Swiss National Science Foundation (Grant No. 315230_192656 to MZW).

## Declaration of competing interest

The authors declare the following financial interests/personal relationships which may be considered as potential competing interests: Marcy Zenobi-Wong reports financial support was provided by 10.13039/100000001Swiss National Science Foundation. If there are other authors, they declare that they have no known competing financial interests or personal relationships that could have appeared to influence the work reported in this paper.

## Figures and Tables

**figs1:**
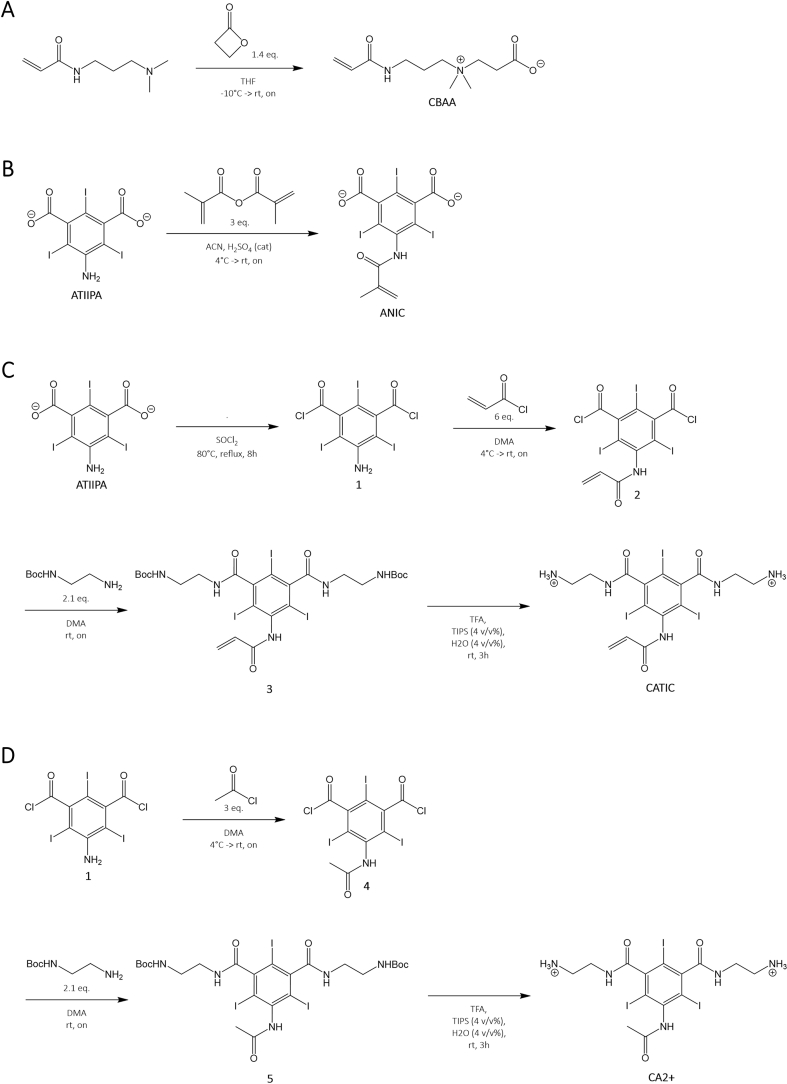
**Figure S1**: **Monomer synthesis:** Overview of the synthesis strategies pursued for the synthesis of the CBAA (A), ANIC (B) and CATIC (C) monomers and the CA2+ reference contrast agent (D).

**figs2:**
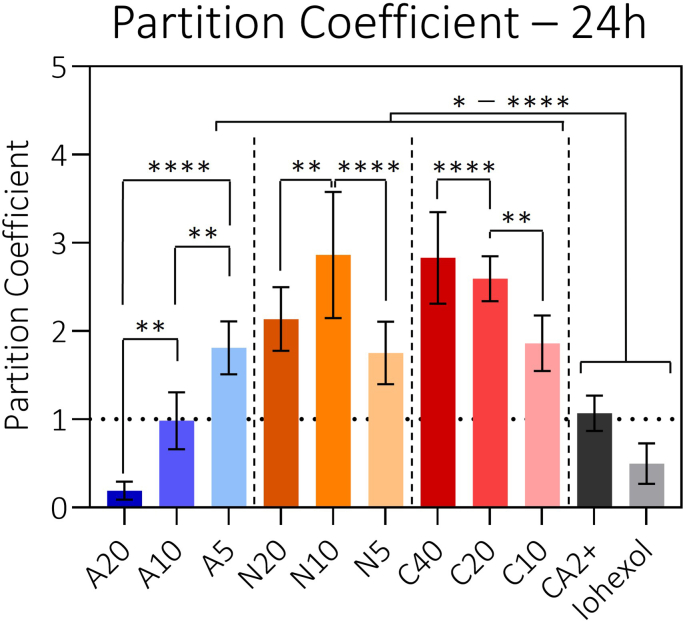
**Figure S2: Partition coefficients after 24h of polymer incubation**: The partition coefficients at the 24h timepoint reveal that most of the investigated polymers show an increased partition coefficient in comparison to CA2+ and iohexol. More concretely, the partition coefficient of CA2+ was measured at slightly above 1, indicating very poor cartilage targeting efficiency. For A20 and iohexol, the partition coefficient was even below 1, indicative of repulsive forces. N=8.

## Data Availability

The data is available at: DOI: 10.3929/ethz-b-000681325, ETH Zurich Research Collection 2024
