# Supplementary material for: Modular iodinated carboxybetaine copolymers as charge-sensitive contrast agents for the detection of cartilage degradation
Source: Mater Today Bio. 2024 Oct 26;29:101302. doi: 10.1016/j.mtbio.2024.101302 (PMC11567940; doi:10.1016/j.mtbio.2024.101302)
Supplement: Multimedia component 1 [file mmc1.docx]

| Sample | A20 | A10 | A5 | N20 | N10 | N5 | C40 | C20 | C10 | CA2+ | Iohexol |
| --- | --- | --- | --- | --- | --- | --- | --- | --- | --- | --- | --- |
| Osmolarity [mOsm] | 308.05 | 308.10 | 308.25 | 308.10 | 308.23 | 308.44 | 308.11 | 308.29 | 308.48 | 312.52 | 310.63 |

**Table S1: Osmolarities for polymer solutions in saline.**
